# Tetramethylpyrazine Retards the Progression and Fibrogenesis of Endometriosis

**DOI:** 10.1007/s43032-021-00813-x

**Published:** 2022-01-31

**Authors:** Shenghui Huang, Fengyi Xiao, Sun-Wei Guo, Tingting Zhang

**Affiliations:** 1 Department of Gynecology, Yueyang Hospital of Integrated Traditional Chinese and Western Medicine, Shanghai University of Traditional Chinese Medicine, Shanghai, China; 2 Department of Gynecology, Shanghai OB/GYN Hospital, Fudan University, Shanghai, 200090 China; 3 Shanghai Key Laboratory of Female Reproductive Endocrine-Related Diseases, Fudan University, Shanghai, China; 4 Shanghai OB/GYN Hospital, Fudan University, 419 Fangxie Road, Shanghai, 200011 China; 5 Department of Gynecology, Yueyang Hospital of Integrated Traditional Chinese and Western Medicine, Shanghai University of Traditional Chinese Medicine, 110 Ganhe Road, Shanghai, 200437 China

**Keywords:** Endometriosis, Epithelial-mesenchymal transition, Fibroblast-to-myofibroblast transdifferentiation, Fibrogenesis, Mouse, Tetramethylpyrazine

## Abstract

The development of more efficacious, non-hormonal therapeutics for endometriosis is still an unmet medical need begging to be fulfilled. Growing evidence indicates that endometriotic lesions are wounds undergoing repeated tissue injury and repair, and, as such, platelets play an important role in lesional progression. Tetramethylpyrazine (TMP), a compound derived from a herb that has been used for thousands of years to combat “blood stasis” in traditional Chinese medicine, is a prescription drug in China for the treatment of cerebrovascular disorders. We tested the hypothesis that TMP can decelerate lesional progression through arresting epithelial-mesenchymal transition (EMT), fibroblast-to-myofibroblast transdifferentiation (FMT), and fibrogenesis. We found in our in vitro experiments that TMP treatment suppresses platelet-induced EMT, FMT, cellular contractility, and collagen production in a concentration-dependent manner. We also showed that in a mouse model of endometriosis, treatment with TMP significantly reduced lesion weight and the extent of lesional fibrosis and improved hyperalgesia, mostly likely through the reduction of lesional aggregation of platelets and the lesional expression of markers of EMT, FMT, and fibrogenesis. In light of our results and in view of its excellent safety profiles, TMP appears to be a promising drug candidate for treating endometriosis.

**Supplementary Information:**

The online version contains supplementary material available at 10.1007/s43032-021-00813-x.

## Introduction

As a major contributing cause of dysmenorrhea, chronic pelvic pain, and infertility, endometriosis is a debilitating gynecologic disease affecting 6–10% of women of reproductive age [1]. Due to the poorly understood pathogenesis and pathophysiology, the clinical management of endometriosis is still a challenge [1]. All existing hormonal therapies interfere with pituitary–gonadal stimulation, induce anovulation and/or a steady hormonal milieu, and reduce or suppress menstrual flow [2]. Given this mechanism of action, these therapies work during treatment but often experience recurrence after discontinuation [2]. In addition, these medications have various side effects and some, such as the GnRH agonists, are restricted by short-term usage [2]. While surgery is efficacious in relieving endometriosis-associated pain [3], it nonetheless carries certain risks of complications, recurrence [4, 5], and elevated risk of adhesion, organ injury, and premature ovarian failure [6–8]. Consequently, more efficacious medical treatment of endometriosis is still an unmet need begging to be fulfilled. Unfortunately, the development of non-hormonal drugs for endometriosis so far has been painfully stagnant [9, 10], and the resultant disappointment is palpable [11]. Remarkably, a recent study reports that nearly 90% of patients with endometriosis are not satisfied with hormonal drugs, and over 95% of patients would have preferred plant-based products [12]. This study provides a strong impetus to search for novel non-hormonal drugs for endometriosis.

Taking cues from the hallmark of ectopic endometrium, which has a defining feature of cyclic bleeding [13], we have previously reported that platelets play important roles in lesional development [14–19], and, as such, antiplatelet therapy appears to be promising [20–22]. Tetramethylpyrazine (TMP), also known as ligustrazine, is a natural alkaloid derived from *Rhizoma Chuanxiong*, and an herb also used in traditional Chinese medicine as a remedy for blood stasis.

TMP is officially listed in the Chinese Pharmacopoeia (2010, expanded and revised edition) for the treatment of cerebrovascular disorders, and its phosphate- and hydrochloride-formulated injections are prescription drugs that are commercially available in China. TMP has been shown to inhibit platelet aggregation [23], oxidative stress [24], and inflammation [25, 26] and attenuate fibrosis [27–29]. In endometriosis, in particular, it has been shown that, as one major ingredient in a concocted herbal formula, TMP suppresses lesional growth, inflammation, and epithelial-mesenchymal transition (EMT) and induces apoptosis [30–32]. Given the excellent safety profiles of TMP, it seems to be an admissible candidate compound for treating endometriosis.

Given the major molecular processes, such as epithelial-mesenchymal transition (EMT), fibroblast-to-myofibroblast transdifferentiation (FMT), and fibrogenesis, which underpin lesional progression are largely known [15, 33, 34], it is unclear whether TMP can decelerate lesional progression through arresting EMT, FMT, and fibrogenesis. This study was undertaken to test the hypothesis that TMP can decelerate lesional progression through arresting EMT, FMT, and fibrogenesis. In this study, we evaluated the effect of TMP on EMT, FMT, and fibrogenesis in endometriosis through in vitro and in vivo experimentations.

## Materials and Methods

### The Cell Line

The endometriotic epithelial cell line (11Z) [35], generously provided by Professor Jung-Hye Choi of Kyung Hee University, Seoul, the Republic of Korea, was established by Professor Anna Starzinski-Powitz and cultured in RPMI 1640 medium (Gibco Laboratories, Life Technologies, Grand Island, NY, USA) as reported previously [15]. The cell line was utilized for quantitative real-time RT-PCR and Western blot under different treatments.

### Human Samples and Cell Culture

The Institutional Ethics Review Board of Yueyang Hospital approved this study (approval number: 2018–082). All tissue samples were collected after written and informed consent from all recruited patients.

Endometriotic tissue samples for in vitro experiments were obtained after informed written consent from 5 cycling premenopausal women (mean age = 32.2 ± 5 years) with laparoscopically and histologically confirmed ovarian endometriomas without other gynecological diseases, who received no antiplatelet or hormonal treatment at least 3 months before the surgery. None of the recruited patients smoked. Previous history of deep venous thrombosis or coagulation disorders or family history was inquired but none reported.

The primary human endometriotic stromal cells (HESCs) were derived and used as reported before [36]. In brief, the endometriotic tissues were rinsed with DMEM/F-12 medium and minced into small pieces of about 1 mm^3^ in size. The minced tissues were digested with 0.2% collagenase II (Sigma, St. Louis, MO, USA) in a shaking bed at 37 °C for 1.5 h, and then separated by filtration through a 76-μm and a 37-μm nylon mesh. The filtrated cells were centrifuged and suspended in DMEM/F-12 medium supplemented with 10% FBS, 100 mg/mL streptomycin, 100 IU/mL penicillin, and 2.5 μg/mL Amphotericin B, and seeded into 25-cm^2^ cell culture flasks and incubated in a humidified atmosphere of 5% CO_2_ in the air at 37 °C. The purity of endometriotic stromal cells was confirmed by immunocytochemistry as we previously reported [37].

HESCs with various treatments were utilized for quantitative real-time RT-PCR, Western blot, cell contractility assay, and collagen assay.

### Preparation of Platelets

Platelets were prepared as described previously [14]. Briefly, 20 mL of peripheral blood samples of healthy volunteers, who had no medication 3 months before donation, were taken from the median cubital vein and blended with 3.2% citric acid acting as an anticoagulant. The platelet-rich plasma (PRP) was acquired by centrifugation for 10 min at 150 g. The pellet portion was divided from the PRP by centrifugation for 10 min at 1,000 g. About 2 × 10^7^ platelets were collected from 1 mL of blood. Finally, a total of∼2 × 10^7^ platelets/mL was put into cell culture dishes. Cells were treated with vehicle (culture medium), or platelets activated with thrombin 0.5 U/mL (T8885, Sigma–Aldrich, St. Louis, MO, USA) equivolumetrically. Thrombin, a potent inducer of platelet activation, was added within 15 min after dilution with culture medium before deactivation as reported previously [38]. This was meant to mimic the situation after cyclic bleeding in eutopic and ectopic endometrium.

### Treatment of Cells

TMP (Sigma, St. Louis, MO, USA), in powdery form, was dissolved in dimethylsulfoxide (DMSO). The concentration of the L-TMP and the H-TMP groups was 25 μMol/mL and 100 μMol/mL, respectively. Before preparation, the cell culture system was diluted by 50-fold, and the final concentration of the control group was 0.1% DMSO. Before processing the cells, they were starved with serum-free medium for 24 h, and then co-cultured with thrombin-activated platelets for 48 (11Z) or 72 h (HESCs).

After treatment with thrombin activated platelets for 48 h, the 11Z cells were treated at 3 concentrations, namely, Control (CTL): PBS + final concentration of 0.1% DMSO, essentially no TMP at all; low-dose TMP (L-TMP): 25 μMol/mL of TMP; high-dose TMP (H-TMP): 100 μMol/mL of TMP. After 48 h of treatment, we evaluated changes in cell morphology, extracted mRNA, and evaluated the gene expression levels in 11Z cells.

After treatment with thrombin-activated platelets for 72 h, the HESCs cells were also treated with the above-mentioned concentrations for 72 h. After that, the cell morphological changes were evaluated, mRNA and proteins were extracted, and mRNA and protein expression levels of FMT-related markers were evaluated, and the function of HESCs was also assessed.

### RNA Isolation and Real-Time RT-PCR

11Z and HESCs cells were co-cultured with activated platelets for 48 or 72 h and sequentially were treated for 48 or 72 h with the above-mentioned concentrations: Control (CTL), low-dose TMP (L-TMP), and high-dose TMP (H-TMP). Using TRIzol (Invitrogen, Carlsbad, CA, USA), the total RNA was separated from all treated cells. The cDNA synthesis was carried out using the PrimeScript™ Reverse Transcriptase (Takara, Takara Bio, Inc., Otsu, Shiga, Japan). The abundance of mRNA was quantified by real-time PCR using SYBR Premix Ex Taq (Takara). Expression values were normalized to the geometric mean of GAPDH measurements and the quantification was done with the method reported previously [39]. The names of genes and their primers are shown in Table 1.

**Table 1:** List of primers used in the real-time RT-PCR analyses

Gene name	Sequence	
GAPDH	Forward	5′-GCACCGTCAAGGCTGAGAAC-3′
	Reverse	5′-TGGTGAAGACGCCAGTGGA-3′
TGF-β1	Forward	5′-TGGAAACCCACAACGAAATC-3′
	Reverse	5′-GGGTTCAGGTACCGCTTCTC-3′
Collagen 1A1 (COL1A1)	Forward	5′-AGGGCCAAGACGAAGACATC-3′
	Reverse	5′-GATCACGTCATCGCACAACA-3′
α-SMA	Forward	5′-GCTTTGCTGGGGACGATGCT-3′
	Reverse	5′-GTCACCCACGTAGCTGTCTT-3′
CCN2	Forward	5′-GGTCAAGCTGCCCGGGAAAT-3′
	Reverse	5′-TGGGTCTGGGCCAAACGTGT-3′

### Western Blot Analysis

To estimate the effect of TMP on the markers of EMT and FMT, 11Z or HESCs cells were co-cultured for 48 or 72 h with activated platelets and sequentially were treated for 48 or 72 h under conditions of CTL, L-TMP, or H-TMP. Furthermore, we evaluated the effect of TMP on changes, if any, in expression levels of proteins involved either in EMT or FMT such as E-cadherin (1:1000, #3195, CST), TGF-β1[40] (1:1000, #Ab92486, Abcam), α-smooth muscle actin (α-SMA, 1:1000, #Ab5694, Abcam), Smad3 (1:1000, #9523, CST), phosphorylated Smad3 (p-Smad3, 1:1000, #9520, CST), and collagen 1A1 (1:1000, #Ab292, Abcam). For total protein extraction, the cells were scraped to extract their proteins in a Radio-Immunoprecipitation Assay (RIPA) buffer (Thermo Fisher Scientific, Pittsburgh, PA, USA). The protein concentration was quantitated with bicinchoninic acid (BCA) protein quantitative analysis kit (P0010S, Beyotime). The protein samples were then loaded on a 10% SDS-PAGE, and transferred to polyvinyl difluoride (PVDF) membranes (Bio-Rad, Hercules, CA, USA). The membranes were incubated overnight at 4 °C with the primary antibodies diluted with PBS, which are listed in Table 2. The diluted antibody solutions were used only once. The membranes were incubated with HRP-labeled secondary antibodies at room temperature for 1 h. Then the signals were developed with enhanced chemiluminescence (ECL) reagents (Pierce, Thermo Scientific, Rockford, IL, USA) and digitized on Image Quant LAS 4000 mini. Image quantification was made with the Quantity One software (Bio-Rad).

**Table 2:** **List** of antibodies used in the Western blot analyses

Antibody name	Catalog number	Host	Isotype	Vendor name and location	Concentration used in Western blot
GAPDH (loading control)	5174	Rabbit	IgG	CST, Boston, MA, USA	1:1000
E-cadherin	3195	Rabbit	IgG	CST	1:1000
TGF-β1	Ab92486	Rabbit	IgG	Abcam, Cambridge, UK	1:1000
α-SMA	Ab5694	Rabbit	IgG	Abcam	1:1000
Smad3	9523	Rabbit	IgG	CST	1:1000
Phosphorylated Smad3 (p-Smad3)	9520	Rabbit	IgG	CST	1:1000
Collagen 1A1 (COL1A1)	Ab292	Rabbit	IgG	Abcam	1:1000

TGF-β1 induces α-SMA synthesis in fibroblasts and stimulates collagen I synthesis [41]. α-SMA is a marker of myofibroblasts and smooth muscle cells [42], and myofibroblasts are the most important effector cell for fibrogenesis. Collagen I is a marker for collagen fibers.

### Collagen Gel Contraction Assay

The cellular contractility is indicative of tissue remodeling capability (e.g., wound contraction). The contractility of cells with various treatments was quantitated by the cellular collagen gel contraction assay kit (CBA-201, Cell Biolabs, San Diego, CA, USA) following the manufacturer’s instructions. HESCs cells were implanted in the collagen gel and cultured three-dimensionally. First co-cultured for 72 h with activated platelets, and then they were treated for 72 h with PBS + final concentrations of 0.1% DMSO (CTL), L-TMP, or H-TMP. Briefly, cells were suspended in the collagen solution (2–5 × 10^6^ cells/mL). The collagen/cell mixture (0.5 mL/plate) was dispensed into 24-well plates (Corning) and incubated for 1 h at 37 °C. Immediately after collagen polymerization, 1 mL of culture medium under various co-culture conditions was put on the top of each collagen gel lattice. After incubation for another 72 h, the collagen gels were gently released with a sterile spatula from the side of the culture dishes. The gels were photographed and then the diameter of each gel surface after release was measured with a vernier caliper at 3, 24, and 48 h, respectively. If the gel surface was oval-shaped, an average was taken from the longest and shortest diameters measured. The difference between the diameter of the well and that of the gel surface denotes the contractility of the cells. The tests were replicated 5 times.

### Collagen Assay

The quantity of soluble collagens produced by cells co-cultured under various conditions was quantified with Sircol soluble collagen assay (S1000, Biocolor, Carrickfergus, UK). The cell culture medium was collected after HESCs were co-cultured with activated platelets for 72 h, and then were co-cultured for 72 h under conditions of the CTL, L-TMP, or H-TMP. The culture medium was gathered and then centrifuged at 12,000 rpm for 10 min to get rid of the particulate materials at the bottom following the manufacturer’s instructions. Since the medium contained serum supplement, low protein binding microcentrifuge tubes (Eppendorf, Hamburg, Germany) were used. The amounts of collagens in the culture medium were measured by the absorbance value at a 570-nm filter. The concentration of collagen was determined by the collagen reference standard curves obtained with a microplate reader (BioTek, Winooski, VT, USA). The experiments were repeated 5 times.

### Animals

Thirty virgin female Balb/C mice, 6 weeks old and ~ 16–18 g in bodyweight, were purchased from the Jiesijie Experimental Animal Company (Shanghai, China). All mice were maintained under controlled conditions with a light/dark cycle of 12/12-h and had access to chows and water ad libitum. All experiments were performed according to the National Research Council’s guidelines[43] and after approval from the institutional experimental animals’ review board of Yueyang Hospital.

### Induction of Endometriosis

A recently reported mouse model of endometriosis by intraperitoneal injection of endometrial pieces with the infusion of substance P (SP) was used [44]. Among the 30 mice, 12 were randomly chosen as donors that supplied uterine tissue fragments, and 18 were recipients who received endometrial tissues from donor mice. Briefly, donor mice were injected with estradiol benzoate (0.2 μg/g bodyweight, Xinyi Chemistry, Shanghai, China) intramuscularly after 1 week of acclimation. They were sacrificed 1 week later, and their uterine tissues harvested. The resultant tissues were then seeded in warm sterile saline in a Petri dish and cut longitudinally with a pair of scissors [45]. The uterine tissues were chopped into small pieces, with a maximal diameter consistently smaller than 1 mm. The fragments were injected into the peritoneal cavity of the recipient mice. To minimize any potential bias, the fragments from 2 donor mice were mixed up and then aliquoted into 3 parts, each injected intraperitoneally to one mouse, each from one of 3 groups. One day before the induction of endometriosis, Alzet osmotic pumps (Model 1004, 0.11 μL/h, DURECT Corporation, Cupertino, CA, USA) packed with SP (100 μg/kg/day; #Ab120170, Abcam) [46], were placed in the nape of the neck in all the recipient mice. Briefly, mice were anaesthetized with 40 mg/kg pentobarbital (China National Medicines Corporation, Ltd, Shanghai, China). A transverse incision about 0.5 cm in length was made on the nape. The skin was divided from muscles towards the back, and an Alzet pump was implanted into the subcutaneous pocket, followed by wound closure. The pump guaranteed controlled and consistent release of contents with a uniform speed.

### Mouse Experiment Protocol

Eighteen recipient mice were randomly separated into 3 equal-sized groups: L-TMP group (TMP dissolved in 50% anhydrous ethanol, 25 mg/kg), H-TMP group (100 mg/kg), and CTL group (50% anhydrous ethanol, no TMP). After 3 weeks of infusion with SP, all recipient mice went through surgery to substitute the old, SP-containing Alzet pumps with new Alzet pumps (Model 2002, 0.5 μL/h, DURECT Corporation) containing different doses of TMP. Eighteen recipient mice were divided into 3 equal-sized groups at random: L-TMP group (TMP dissolved in 50% anhydrous ethanol, 25 mg/kg), H-TMP group (100 mg/kg), and CTL group (50% anhydrous ethanol, no TMP). Two weeks after the treatment, all mice were sacrificed by cervical dislocation and evaluated.

### Lesion Measurement and the Hotplate Test

For all mice, the bodyweight and hotplate latency were measured on days 0, 7, 14, 21, and 35 days after the induction of endometriosis. After sacrifice, the abdominal cavity was opened up right away and examined carefully. All discernable endometriotic lesions were removed and weighed (dry weight) as reported earlier [44]. All endometriotic lesions were then prepared for histological confirmation and immunohistochemical analysis, along with Masson trichrome staining to evaluate the extent of lesional fibrosis.

Since rodents with induced endometriosis and women with endometriosis display central sensitization, and in view of the fact that mice are not vocal about their pain severity [16, 21, 44, 47–50], we assessed the hotplate latency of mice as a surrogacy measure for pain. A commercially available Hot Plate Analgesia Meter (Model BME-480, Institute of Biomedical Engineering, Chinese Academy of Medical Sciences, Tianjin, China) was employed for hotplate test, as previously reported [47, 48].

### Immunohistochemistry

Tissue samples were fixed using 10% formalin (w/v) and paraffin-embedded. Serial 4-μm sections were acquired from each block, with the initial resultant slide being stained with hematoxylin and eosin to confirm the pathologic diagnosis, and the following slides for immunohistochemistry (IHC) analysis for CD41(1:100, #Ab33661, Abcam), TGF-β1 (1:100, #MAB240, Novus Biologicals), neurokinin receptor 1 (NK1R) (1:100, #Ab183713, Abcam), α-SMA (1:200, #Ab5694, Abcam), and collagen I (1:100, #Ab292, Abcam). The list of antibodies used in IHC is shown in Table 3. We note that CD41 is for a marker for activated platelets, which have been shown to be involved in the angiogenesis and fibrogenesis of endometriosis [14, 15]. NK1R has been shown to accelerate lesional progression through neuropeptide substance P [51].

**Table 3:** List of antibodies used in the immunohistochemistry analyses

Antibody name	Catalog number	Host species	Isotype	Vendor name and location	Concentration used in immunohistochemistry
CD41	Ab33661	Rat	IgG1	Abcam, Cambridge, UK	1:100
TGF-β1	MAB240	Mouse	IgG1	Novus Biologicals, Littleton, CO, USA	1:100
α-SMA	Ab5694	Rabbit	IgG	Abcam	1:200
Collagen 1A1 (COL1A1)	Ab292	Rabbit	IgG	Abcam	1:100
NK1R	Ab183713	Rabbit	IgG	Abcam	1:100

Five slides from the same mouse were utilized for each marker. For negative controls, tissue samples were incubated with a mouse or rabbit serum rather than primary antibodies. The representative figures of these controls can be found in Supplementary Figure S1.

Standard deparaffinization and rehydration procedures were performed, as reported before [21]. For antigen retrieval, the slides were heated at 98 °C for a total of 30 min in citrate buffer (pH6.0) and then cooled to room temperature. The slides were next incubated with the primary antibodies, diluted with PBS, at 4 °C overnight. The slides were rinsed with PBS, and then the horseradish peroxidase–labeled secondary antibody Detection Reagent (Sunpoly-HII; BioSun Technology Co, Ltd, Shanghai, China) was added and incubated for 30 min at room temperature. The bound antibody complexes were stained with diaminobenzidine until suitable for microscopic examination and subsequently counterstained with hematoxylin for 30 s and mounted. Images were taken with the microscope (Olympus BX53; Olympus, Tokyo, Japan) fitted with a digital camera (Olympus DP73; Olympus). For each marker, 3–5 images were selected randomly at 400 × magnification for each sample to obtain a mean optical density value via the software Image Pro-Plus 6.0 as reported previously [14, 52]. To maintain objectivity, the group identity of the sample to be evaluated was blinded to the evaluator.

### Masson Trichrome Staining

Masson trichrome staining was employed for the assessment and semi-quantification of collagen fibers in endometriotic tissue samples. Tissue sections were deparaffinized in xylene and rehydrated in a graded alcohol series then were immersed in Bouin’s solution at 37 °C for 2 h as reported previously [33]. Sections were stained with the Masson’s Trichrome Staining kit (Baso, Wuhan, China) following the manufacturer's instructions. In general, 3–5 images containing the glandular epithelium were taken under a 20-power objective lens, covering the endometriotic lesions as much as possible. The percentage of the collagen fiber area stained in blue relative to the entire field of the ectopic endometrium, analyzed by the Image Pro-Plus 6.0, was taken as the proportion of fibrotic content in the lesion.

### Statistical Analysis

The comparison of distributions of continuous variables between or among two or more groups was made with Wilcoxon’s and Kruskal’s tests, respectively. To minimize the problem of multiple testing and to use the data more efficiently (especially when in the evaluation of gene expression for 11Z cells as *n* = 3 in each group), linear regression analyses were used to determine whether there is a dose–response effect on gene/protein expression levels, collagen production and cellular contractility with the concentration of TMP (and time of measure for contractility) as co-variable(s). This is because we were looking to see whether there is a concentration-dependent relationship between the concentration and the gene/protein expression levels (or collagen production or contractility, whichever is of interest). As such, we could view the concentration as the independent variable, the expression level as the dependent variable, and then use the linear regression (which is equivalent to ANOVA when there is only one variable). The normality assumption was checked by plotting the regression residuals using a Q-Q plot. The data were either square-root and/or log transformed to enhance normality. We note that the univariate linear regression, which is equivalent to ANOVA, is known to be robust to the departure of normality assumption [53, 54]. In case when the normality assumption was in doubt, we also performed a randomization test [55] using the regression slope estimate as the statistic, and the estimated *p* value was calculated based on 1 million permutations.

The choice of sample sizes in the mouse study was based on our previous experience with the model. No prior sample size estimation was calculated. *p* values of < 0.05 were deemed statistically significant. All computations were made with R 4.1.1 [56].

## Results

### TMP Suppresses Platelet-Induced EMT in Endometriotic Epithelial Cells

**Fig. 1:**
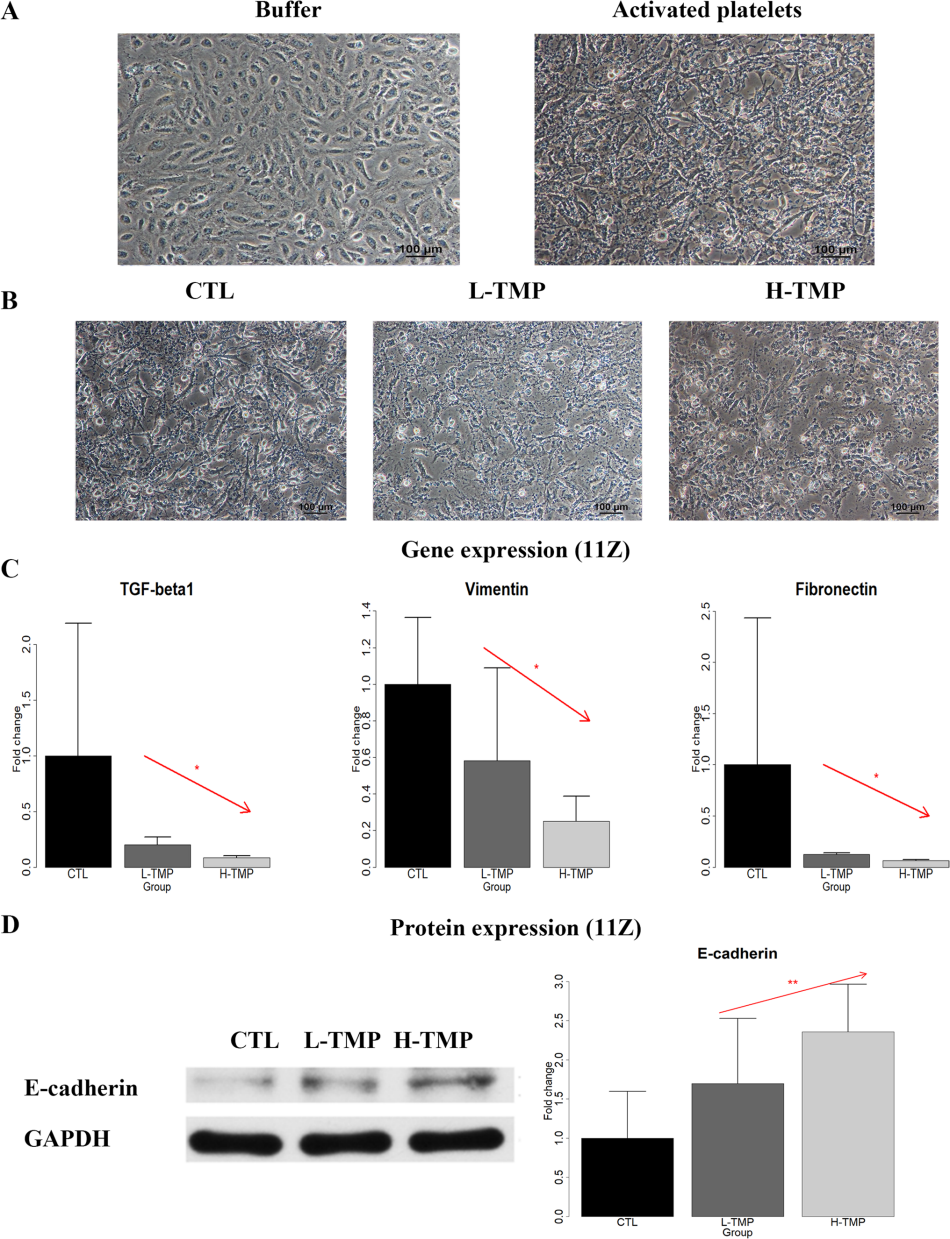
The effect of TMP treatment on morphological and molecular changes in endometriotic epithelial cells**. A** Representative micrographs of endometriotic epithelial cells (11Z) treated with buffer or activated platelets (by thrombin) for 48 h. Scale bar = 100 μm. **B** Representative micrographs of 11Z cells first co-cultured with activated platelets for 48 h, and then treated either with PBS + final concentration of 0.1% DMSO (Control or CTL), TMP with a final concentration of 25 μMol/mL (low-dose TMP or L-TMP), or TMP with a final concentration of 100 μmol/mL (high-dose LMP, or H-TMP) for 48 h. Scale bar = 100 μm. **C** Relative fold change in gene expression of TGF-β1, vimentin, and fibronectin in 11Z cells treated with the abovementioned 3 conditions for 48 h (*n* = 3). Values were normalized to GAPDH expression. **D** Left panel: Detection of protein levels of E-cadherin by immunoblotting of 11Z cells treated with the indicated conditions as in **B** for 48 h. Right panel: Relative fold change of protein levels of E-cadherin in 11Z cells treated as in **B** for 48 h (*n* = 5). In panels **C** and **D**, linear regression analysis was used, with the TMP concentration as the co-variable. The red arrow indicates the linear trend of concentration dependency. Symbols of statistical significance levels: *: *p* < 0.05; **: *p* < 0.01. Data are represented in means ± SDs

Activated platelets have been shown to induce EMT, and FMT in endometriotic cells [15]. Therefore, we first investigated whether TMP could suppress platelet-induced changes in morphology, expression of genes involved in EMT of endometriotic epithelial cells (11Z). We co-cultured 11Z cells with buffer (PBS) or activated platelets, induced by thrombin, for 48 h, and confirmed that activated platelets induced dramatic morphological changes suggestive of EMT, with the morphology changed from round-shaped to spindle-like and became dispersed (Fig. 1A).

We also found that treatment with low-dose (L-TMP) or high-dose TMP (H-TMP) attenuated platelet-induced morphological changes of EMT in 11Z (Fig. 1B). Based on linear regression analysis using the concentration of TMP as the co-variable, we found that, consistently, treatment with L-TMP and H-TMP concentration-dependently suppressed platelet-induced activation of TGF-β1, vimentin and fibronectin in endometriotic epithelial cells (all *p* values < 0.030, all *R*^*2*^ ≥ 0.51; Fig. 1C; The randomization test yielded all *p* values < 0.033). In addition, Western blot analysis confirmed that the treatment of TMP concentration-dependently increased platelet-suppressed protein expression of E-cadherin (*p* = 0.0063 by linear regression, *R*^*2*^ = 0.45; Fig. 1D; the randomization test gave the estimated *p* value of 0.0068). Thus, TMP treatment concentration-dependently suppressed EMT and EMT-like phenotypes induced by activated platelets in endometriotic epithelial cells.

### TMP Suppresses Platelet-Induced FMT in Endometriotic Stromal Cells

**Fig. 2:**
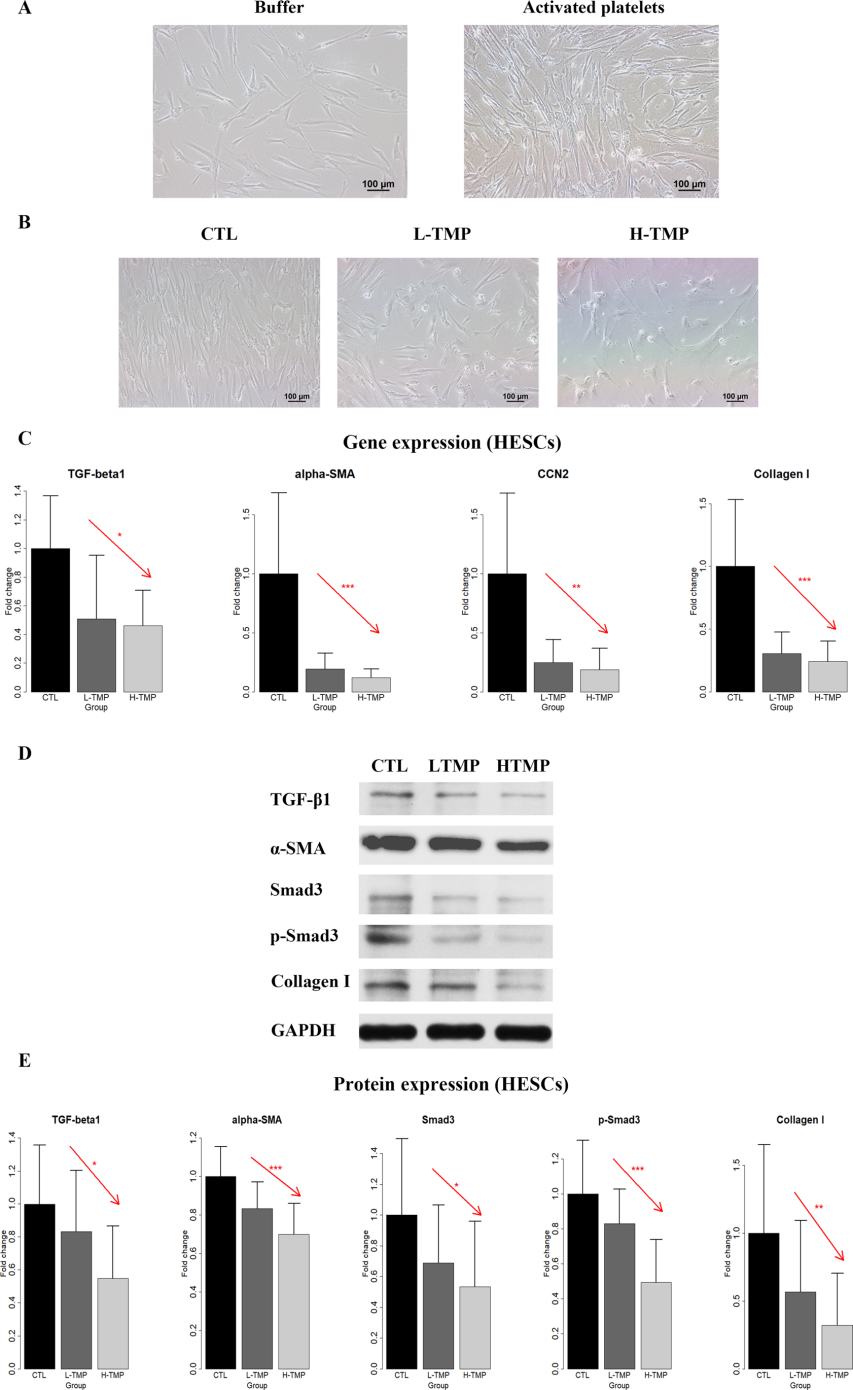
The effect of TMP treatment on morphological and molecular changes in primary endometriotic stromal cells. **A** Representative micrographs of human primary endometriotic stromal cells (HESCs) treated with buffer or activated platelets (by thrombin) for 72 h. Scale bar = 100 μm. **B** Representative micrographs of HESCs first co-cultured with activated platelets for 72 h, and then treated either with PBS + final concentration of 0.1% DMSO (control or CTL), TMP with a final concentration of 25 μMol/mL (low-dose TMP or L-TMP), or TMP with a final concentration of 100 μmol/mL (high-dose LMP, or H-TMP), for 72 h. Scale bar = 100 μm. **C** Relative fold change in gene expression levels of TGF-β1, α-SMA, CCN2, and collagen I in HESCs treated with the 3 conditions mentioned above for 72 h (*n* = 5). Values are normalized to GAPDH expression. **D** Detection of protein levels of TGF-β1, α-SMA, Smad3, phosphorylated Smad3 (p-Smad3), and collagen I by immunoblotting of lysates of HESCs treated with the indicated conditions as in **B**. **E** Relative fold change of protein expression levels of TGF-β1, α-SMA, Smad3, p-Smad3, and collagen I in HESCs treated with the indicated conditions as in **B** for 72 h (*n* = 8). In panels **C** and **E**, linear regression analysis was used, with the TMP concentration as the co-variable. The red arrow indicates the linear trend of concentration dependency. Symbols of statistical significance levels: *: *p* < 0.05; **: *p* < 0.01; ***: *p* < 0.001. Data are represented in means ± SDs

We next investigated whether TMP could suppress platelet-induced changes in morphology, change the expression of genes or proteins involved in FMT as well as functions of human endometriotic stromal cells (HESCs). We co-cultured HESCs with buffer or activated platelets for 72 h and found that activated platelets induced conspicuous morphological changes reminiscent of myofibroblast activation that is consistent with FMT, as these cells went from a spindle-like morphology to a thinner and more elongated morphology suggestive of muscle fibers and further dispersed (Fig. 2A). However, treatment with TMP also completely abolished platelet-induced morphological changes, as the HESCs treated with platelets and low- or high-dose TMP displayed a spindle-like morphology as compared with a thinner and more elongated morphology in the CTL group (in which the HESCs were first treated with activated platelets for 72 h) (Fig. 2B).

Next, we evaluated the expression of genes known to be involved in FMT in HESCs and analyzed data by linear regression using the concentration of TMP as the co-variable. We found that the treatment of TMP concentration-dependently suppressed platelet-induced gene expression levels of α-SMA, CCN2, and collagen I in HESCs (all *p* values ≤ 0.036, all *R*^*2*^ ≥ 0.30; Fig. 2C; all *p* values < 0.0377 by randomization test). Consistently, Western blot analysis confirmed that the treatment of TMP concentration-dependently suppressed platelet-induced protein expression of TGF-β1, α-SMA, p-Smad3, and collagen I (all *p* values ≤ 0.040 by linear regression, all *R*^*2*^ ≥ 0.18; Fig. 2D; all *p* values < 0.0409 by the randomization test). Thus, these results indicate that treatment with TMP suppresses FMT induced by platelets in HESCs.

### TMP Abrogates Platelet-Induced Contractility and Collagen Production in Endometriotic Stromal Cells

**Fig. 3:**
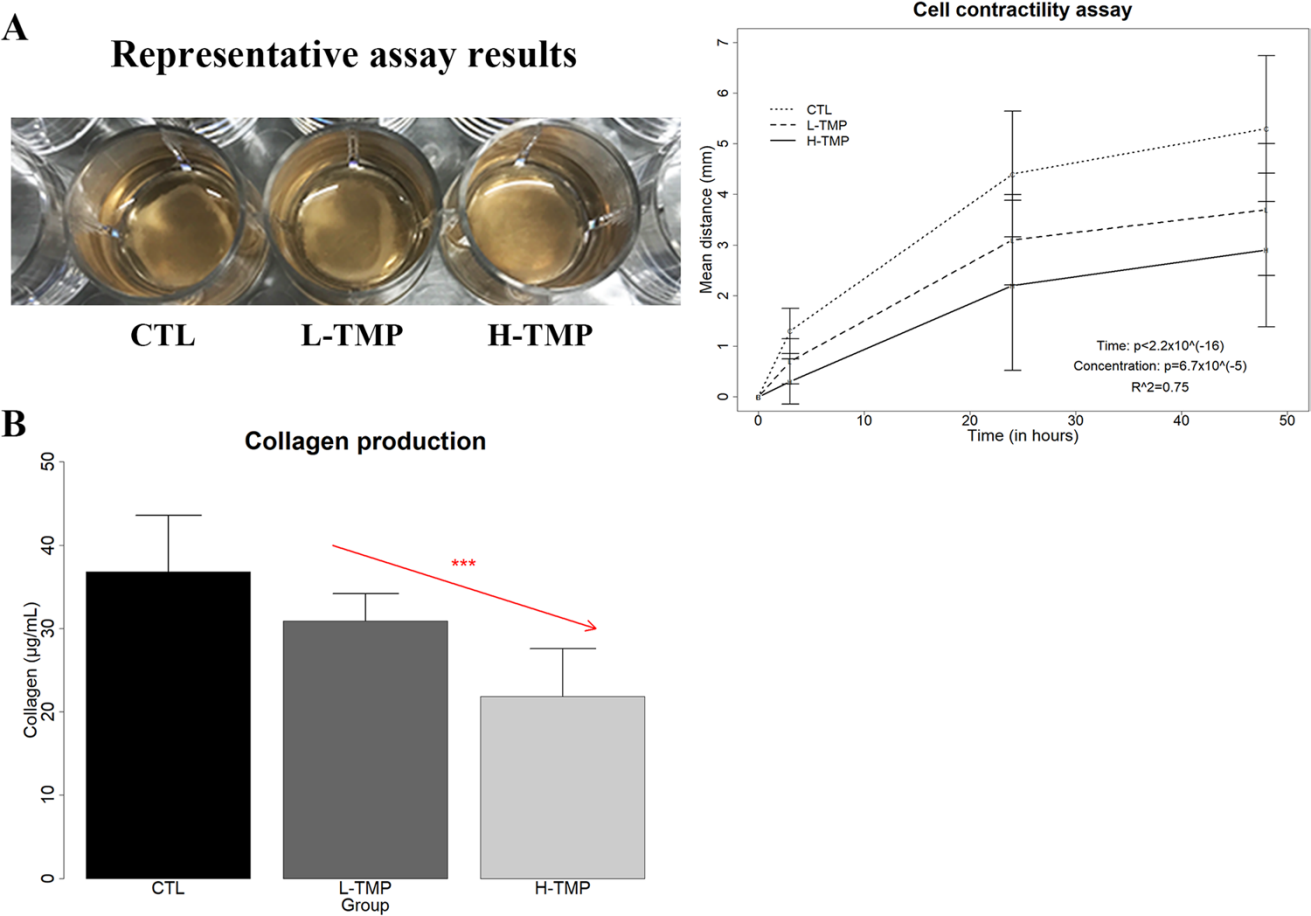
The effect of TMP treatment on cellular contractility and collagen production in primary endometriotic stromal cells. **A** Left: the representative results of collagen gel contraction assay for HESCs first co-cultured for 72 h with activated platelets, and then treated with the indicated conditions for 48 h. Right: Summary of the contractility results, in terms of diameter of the gel surface, for HESCs measured at 0. 3, 24, and 48 h, respectively (*n* = 5). **B** The soluble collagen secreted by HESCs first co-cultured for 72 h with activated platelets, and then treated with the indicated conditions for 72 h (*n* = 5). Linear regression analysis was used, with the TMP concentration as the co-variable. The red arrow indicates the linear trend of concentration dependency. Symbols of statistical significance levels: ***: *p* < 0.001. Data are represented in means ± SDs

The transdifferentiation of fibroblasts to myofibroblasts is characterized by increased cellular contractility and collagen production, which can manifest as tissue fibrosis. To see whether TMP could abrogate the FMT-like functional changes induced by activated platelets, we evaluated the contractility of HESCs co-cultured with activated platelets and treated with either vehicle (CTL), L-TMP, or H-TMP. While the contractility increased significantly with treatment time (*p* < 2.2 × 10^–16^), treatment with TMP concentration-dependently attenuated the increase in contractility of HESCs induced by activated platelets (*p* = 6.7 × 10^–5^ by linear regression, *R*^*2*^ = 0.75; Fig. 3A). Moreover, treatment with TMP concentration-dependently reduced platelet-induced production of soluble collagens in HESCs (*p* = 0.0006 by linear regression, *R*^*2*^ = 0.60; Fig. 3B; *p* = 0.00033 by the randomization test).

In conjunction with the results presented above, we hence conclude that TMP abrogates platelet-induced myofibroblast activation in endometriotic stromal cells, resulting in significantly reduced cellular contractility and collagen production.

### Treatment with TMP Decreases Lesion Weight and the Extent of Lesional Fibrosis and Improves Hyperalgesia

**Fig. 4:**
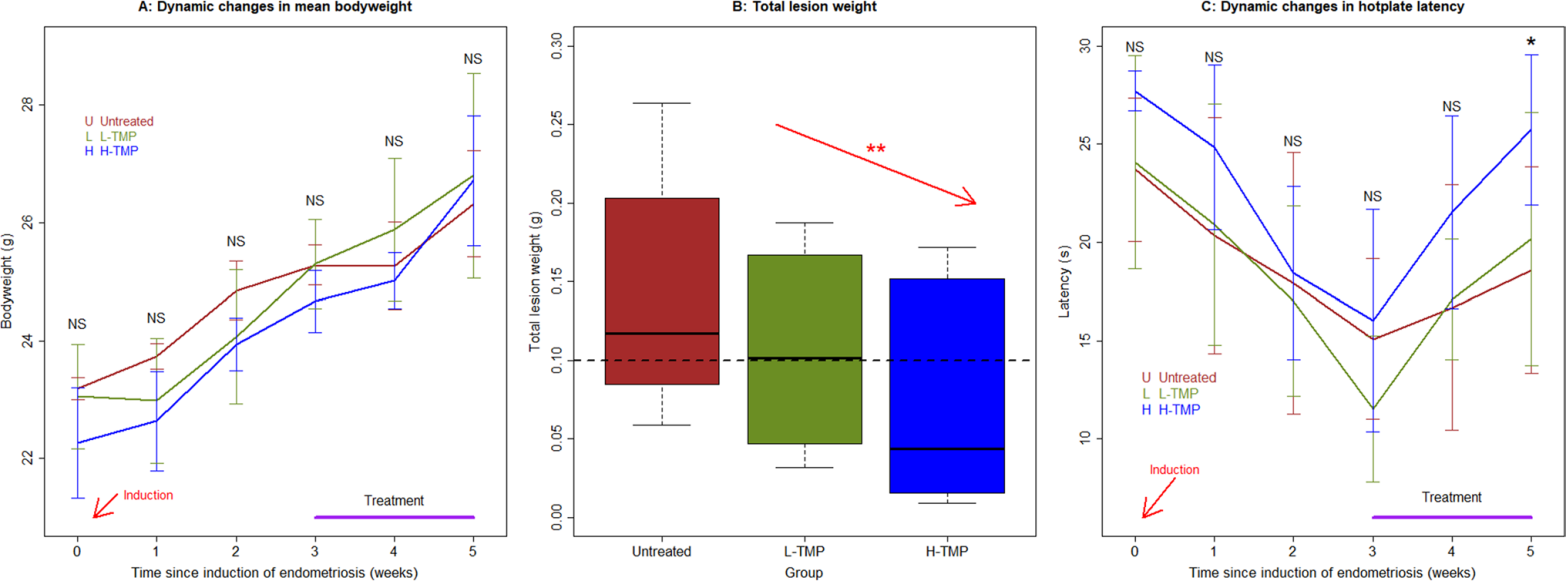
Dynamic changes in the mean bodyweight, lesion weight, and hotplate latency. **A** Dynamic changes in the mean bodyweight in different groups of mice.** B** Boxplot showing the total lesion weight in different treatment groups. The dashed line represents the median value of all mice. Linear regression analysis was used, with the TMP concentration as the co-variable. The red arrow indicates the linear trend of concentration dependency. **C** Dynamic changes in the mean hotplate latency. In panels **A** and **C**, the data are represented by the means ± SDs. Symbols of statistical significance levels: **: *p* < 0.01; NS: not statistically significant (*p>* 0.05). And the statistical significance levels refer to the difference among the entire 3 groups (Kruskal’s test)

In light of the in vitro findings presented above, we conducted an in vivo experiment to evaluate the effect of TMP treatment in a mouse model of endometriosis. We found that TMP is well-tolerated in treated mice, and no mouse died during the entire experimental period. There was no difference in bodyweight among the three groups of mice before the induction of endometriosis (*p* = 0.10 by Kruskal’s test; Fig. 4A), suggesting that there was no difference in bodyweight among the 3 groups. Multiple linear regression analysis incorporating the TMP dosage, time since induction, and the baseline bodyweight indicated that TMP treatment had no effect on bodyweight 1, 2, and 3 weeks after induction (all *p* values > 0.25 by linear regression, *R*^*2*^ ≥ 0.33; Fig. 4A). Multiple linear regression analysis of bodyweight after the start of treatment incorporating the TMP dosage, time of measurement, and the bodyweight before the treatment indicated that TMP treatment had no effect on bodyweight 1 and 2 weeks after treatment (all *p* values > 0.25 by linear regression, *R*^*2*^ ≥ 0.33; Fig. 4A), suggesting that TMP did not impact on growth.

Treatment with TMP resulted in significantly lower lesion weight than the control mice in a dose-dependent manner (*p* = 0.0039 by linear regression, *R*^*2*^ = 0.61; Fig. 4B). The average weight in L-TMP and H-TMP groups was reduced by 24.8% and 48.5%, respectively, compared with that of the untreated mice (Fig. 4B), suggesting that the TMP treatment restrained lesional growth and progression.

Before the induction of endometriosis or prior to the TMP treatment, there was no difference in hotplate latency among the 3 groups (both *p* values = 0.22 by Kruskal’s test, respectively; Fig. 4C), and there was a significant and progressive reduction in latency 1, 2, and 3 weeks after the induction of endometriosis (*p* = 0.012, *p* = 1.5 × 10^–5^, and *p* = 7.6 × 10^–6^ by Wilcoxon’s test, respectively; Fig. 4C), as we reported previously [20, 57]. Multiple linear regression analysis incorporating the TMP dosage and the baseline hotplate latency indicated that there was no difference in hotplate latency 1, 2, and 3 weeks after induction among the 3 groups as the treatment started only thereafter (all *p* values > 0.31 by linear regression, *R*^*2*^ ≥ 0.30; Fig. 4C). While no difference in hotplate latency was found 1 week after treatment (*p* = 0.20 by Kruskal’s test; Fig. 4C), multiple linear regression analysis incorporating the TMP dosage and the latency before the treatment indicated that TMP treatment resulted in significantly longer latency 2 weeks after the treatment than the untreated mice in a dose-dependent manner (*p* = 0.025 by linear regression, *R*^*2*^ = 0.46; Fig. 4C), and the after-treatment latency was positively associated with the before-treatment latency (*p* = 0.034).

### TMP Suppresses the Aggregation of Platelets, the Expression of TGF-β1, NK1R, α-SMA, and Collagen I, and the Extent of Fibrosis in Mouse Endometriotic Lesions

We also performed immunostaining of CD41 (for platelet aggregation), TGF-β1, NK1R, α-SMA, and collagen I. Moreover, we evaluated the extent of lesional fibrosis through Masson trichrome staining.

**Fig. 5:**
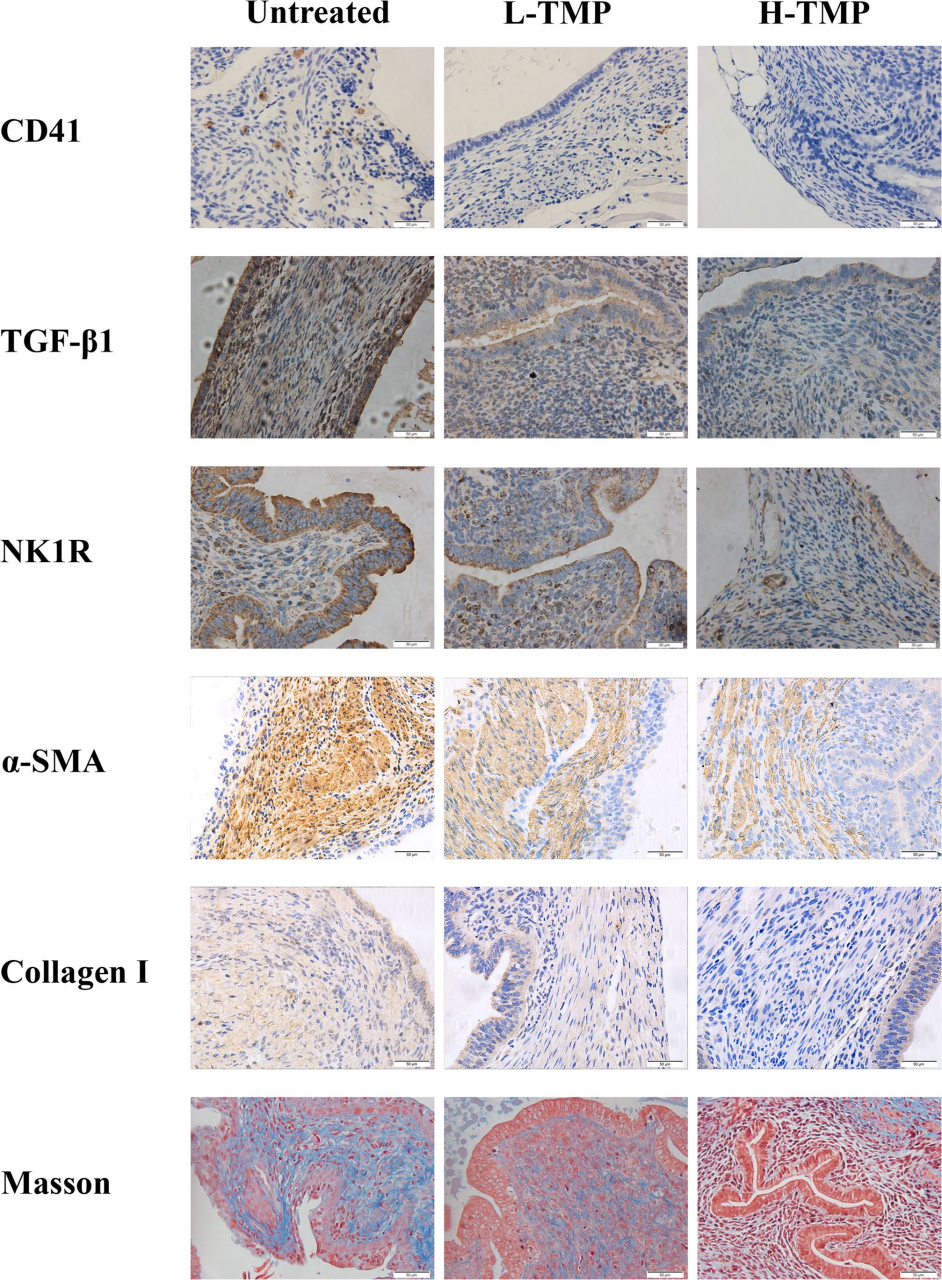
Representative photomicrographs of immunostaining results in endometriotic lesions from 3 groups of mice. Different rows show different staining markers as indicated. Different columns represent mice without treatment of TMP **(**untreated), treated with TMP low-dose (L-TMP), or treated with high-dose TMP (H-TMP). In Masson trichrome staining, the collagen fibers in lesions were stained in blue. In all figures, Magnification: × 400. Scale bar = 50 μm

We found that platelets (CD41 +) aggregated mostly in the stromal component of endometriotic lesions, and some could be found around epithelial cells (Fig. 5). TGF-β1 staining was seen primarily in glandular epithelial cells and was localized in the cytoplasm (Fig. 5). NK1R staining was seen mostly in the cytoplasm and membranes in endometriotic epithelial cells. α-SMA and collagen I staining were primarily seen in the cytoplasm in endometriotic stromal cells (Fig. 5).

**Fig. 6:**
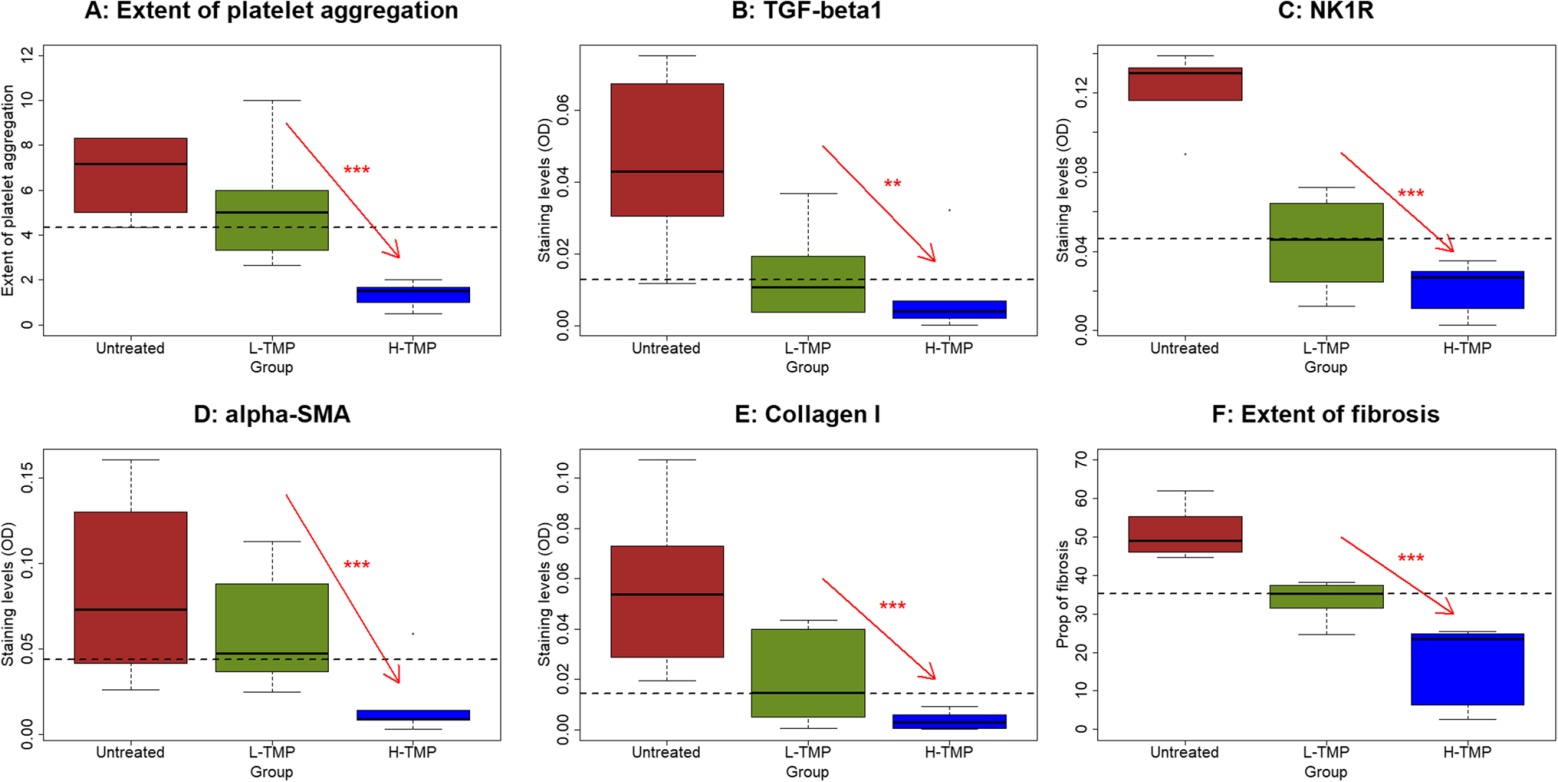
Summary results of immunohistochemical staining of CD41, TGF-β1, NK1R, α-SMA, collagen I, as well as Masson trichrome staining in endometriotic lesions from 3 groups of mice. Boxplots showing the density of CD41 + platelets (**A**), lesional staining of TGF-β1 (**B**), NK1R (**C**), α-SMA (**D**), and collagen I (**E**), and the extent of lesional fibrosis as evaluated by Masson trichrome staining (**F**). Linear regression analysis was used, with the TMP concentration as the co-variable. The red arrow indicates the linear trend of concentration dependency. Symbols of statistical significance levels: ***: *p* < 0.001. Untreated: mice receiving no TMP treatment; L-TMP: mice receiving low-dose TMP treatment; H-TMP: mice receiving high-dose TMP treatment

Through regression analysis, we found that TMP treatment significantly and dose-dependently reduced the extent of lesional aggregation of platelets, lesional staining of TGF-β1, NK1R, α-SMA, and collagen I (*p* = 3.3 × 10^–6^, *R*^*2*^ = 0.75, *p* = 0.0013, *R*^*2*^ = 0.49, *p* = 1.1 × 10^–6^, *R*^*2*^ = 0.78, *p* = 0.0003, *R*^*2*^ = 0.57, *p* = 9.2 × 10^–5^, *R*^*2*^ = 0.62, respectively, all by linear regression; Fig. 5 and 6).

Consistent with the reduction of lesion weight, the extent of lesional fibrosis also was reduced in a dose-dependent fashion (*p* = 7.2 × 10^–7^ by linear regression, *R*^*2*^ = 0.79; Fig. 5 and 6).

### The Extent of Lesional Fibrosis Correlates with the Lesion Weight, Hotplate Latency, and Lesional TGF-β1, NK1R, α-SMA, and Collagen I Expression Levels

**Fig. 7:**
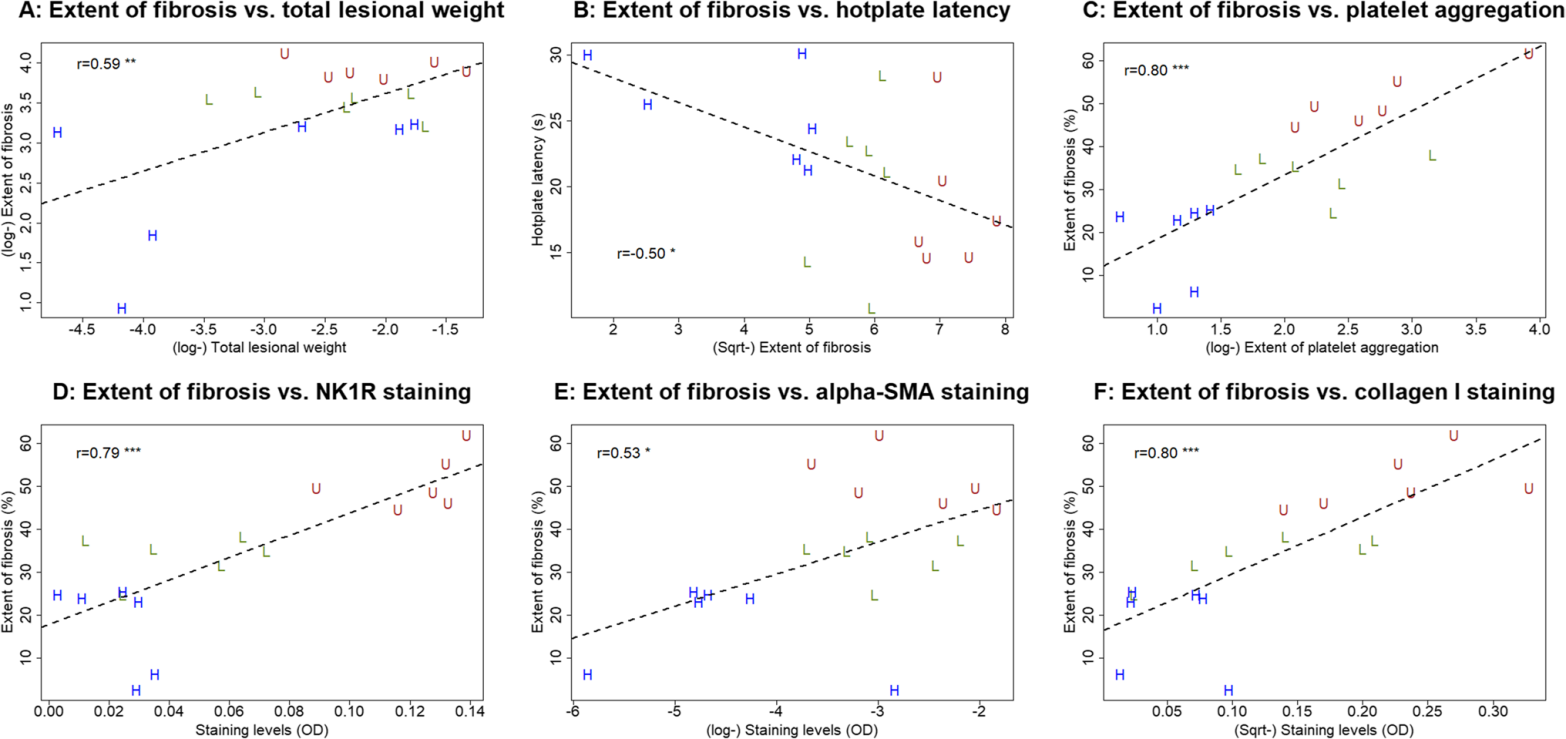
Correlation between lesion weight, or immunohistochemistry staining and fibrotic content of endometriotic lesions. Scatter plots showing the relationship between the extent of lesional fibrosis and the lesion weight (**A**), the hotplate latency (**B**), the extent of platelet aggregation (**C**), lesional staining of NK1R (**D**), α-SMA (**E**), and collage I (**F**). Each dot represents 1 data point from 1 mouse. The dashed line is the regression line. The number shown in each figure is the Pearson’s correlation coefficient, followed by a symbol indicating its statistical significance level. Symbols of statistical significance levels: **: *p* < 0.01; *** *p* < 0.001. log: log-transformed; Sqrt: square root transformed. OD: optical density

The extent of lesional fibrosis correlated positively with the lesion weight (*r* = 0.59, *p* = 0.0096; Fig. 7A) but negatively with the hotplate latency (*r* =  − 0.50, *p* = 0.033; Fig. 7B). While it correlated marginally with the lesional TGF-β1 staining (*r* = 0.45, *p* = 0.062), it correlated positively with the extent of platelet aggregation in lesions (*r* = 0.80, *p* = 7.0 × 10^–5^; Fig. 7C), and lesional staining levels of NK1R (*r* = 0.79, *p* = 8.9 × 10^–5^; Fig. 7D), α-SMA (*r* = 0.53, *p* = 0.024; Fig. 4E), and collagen I (*r* = 0.80, *p* = 7.6 × 10^–5^; Fig. 7F).

## Discussion

In this study, we have provided in vitro evidence to show that TMP treatment suppresses platelet-induced EMT, FMT, cellular contractility, and collagen production. We also have shown that, in a mouse model of endometriosis, treatment with TMP significantly reduced lesion weight and the extent of lesional fibrosis and alleviated hyperalgesia, mostly likely through the reduction of lesional aggregation of platelets and the lesional expression of TGF-β1, NK1R, α-SMA, and collagen I. Our findings indicate that TMP can be an excellent drug candidate for treating endometriosis.

Given the growing evidence for the role of platelets in driving the progression of endometriosis [14, 15, 18, 19, 21] and for the hypercoagulant state in women with endometriosis [58–60], it is not surprising to see that platelet depletion and antiplatelet treatment effectively suppress lesion growth in mouse with induced endometriosis [14, 20, 49]. In addition, many compounds with therapeutic potentials in treating endometriosis [61], such as andrographolide [62, 63], apigenin [64], scutellarin [50], tanshinone IIA [21, 65], and wogonin [66], all have the commonality of being antiplatelet or anti-thrombotic [67–71]. Even some drugs that are known to be therapeutic for endometriosis or have therapeutic potentials, such as danazol [72] and valproic acid [73, 74], are also antiplatelet/anti-thrombotic [75, 76]. Many of these compounds also have been shown to be anti-fibrotic [77–81].

This commonality of antiplatelet/anti-thrombotic shared by these potential therapeutics for endometriosis is, in our opinion, not just a coincidence, but, rather, due primarily to the well-known role of platelets in tissue repair, as platelets are the first (anucleated) cells going to the wounding site, serving their duties in hemostasis and recruiting other immune cells. Platelets contain mRNA and translational machinery, and, as such, they are capable of synthesizing proteins [82]. Equipped with a complex system of granules, platelets, once activated, can release up to 300 bioactive molecules, including well-known profibrotic factors such as TGF-β1 and PDGF, two potent molecules that can work in concert to promote fibrogenesis [83].

Aside from their well-elucidated role in hemostasis and thrombosis, a growing body of evidence in the last decade also indicates their role in inflammation and immune responses [84, 85]. This is due, in no small part, to the fact that injuries require both an efficient hemostasis and an inflammatory immune response against invading pathogens, hence the close linkage between inflammatory and thrombotic processes is.assumed to have an evolutionary origin [86, 87].

Platelets within the lesional microenvironment may also regulate immune function lesions, conferring a microenvironment that is conducive to lesion growth. Indeed, platelet-derived TGF-β1 is reported to suppress the expression of natural killer (NK) group 2, member D (NKG2D), an activating surface receptor, on NK cells, resulting in reduced cytotoxicity in women with endometriosis [88]. In fact, platelets are shown to impair NK cell reactivity and function in endometriosis through multiple mechanisms [16]. Platelet-derived ectosomes may also impair NK cell function [89].

TGF-β1 is by far the most potent profibrotic mediator known in humans, which was initially discovered in platelets and is released copiously also by platelets [90]. It is a key player in tissue repair, is normally upregulated following tissue injury, and plays a pivotal role in the production of extracellular matrix. Long been implicated in fibrosis, TGF-β1 has also been implicated in the lesional progression and fibrogenesis [15, 18, 19]. There are two distinct forms of TGF-β1: a latent and an active form. Platelets are a rich source of TGF-β1 but also contain activators of latent TGF-β1 [91, 92]. Conversely, TGF-β1 also enhances platelet aggregation [93]. Conceivably, platelets are activated following contact with the damaged endothelium within lesions during menstruation and subsequently release latent TGF-β. Platelets can convert latent TGF-β1 to its active form which can stimulate nearby endometriotic stromal/fibroblasts and trigger the fibrotic process. Moreover, TGF-β1 may enhance platelet activation leading to further TGF-β1 release and vascular damage, forming a vicious circle in lesions that is difficult to break.

We chose NK1R for IHC analysis in mouse experiments for two reasons. First, NK1R has been shown to play a critical role in accelerating lesional progression [51, 94]. Second, NK1R is involved in substance P (SP) mediated platelet aggregation [95]. Our results indicate that TMP treatment dose-dependently reduced the lesional expression of NK1R, which also could be one additional reason for the lesional arrest, especially fibrogenesis. Since the precise role of SP/NK1R in coagulation is an active area for research [96], future studies are needed to delineate their roles in lesional progression.

Our data are consistent with the report that TMP suppresses platelet aggregation [23], downregulates the TGF-β1 pathway [97, 98], and suppresses EMT [99]. In addition, our data have shown that TMP treatment reduces the lesional expression of NK1R, the receptor for neuropeptide SP, which has been shown to be involved in the acceleration of lesional progression [51, 94].

It is well known that there is a very high attrition rate in drug research and development (R&D): from discovery to the successful regulatory approval for marketing, over 99% of compounds do not make it to the final stage of marketing approval [100]. Drug R&D for endometriosis is no exception [9]. Many compounds failed simply because of unacceptable safety profiles, or inferior efficacy, or both. Given this grim reality, one seemingly short-cut would be the screening of compounds derived from medicinal herbals, which often have a good safety profile. TMP fits this bill nicely: its phosphate- and hydrochloride-formulated injections have been used in China for over 30 years and are known to have a good safety profile.

Nonetheless, a recent paper summarized various, though uncommon, side effects of TMP hydrochloride injection, such as allergic reaction, fever, palpitation, choking sensation, hypotension, headache, gastrointestinal reactions, acute hemolytic uremia, convulsions, edema, and other adverse reactions, that have been reported in various Chinese journals during 2000 − 2013 [101]. However, it is unclear whether these side effects are attributable to the compound itself, or formulation, or problems in manufacturing. It should be noted that adverse events arising from the injection formulations made from the traditional Chinese medicine herbs/extracts are particularly a perennial and pervasive problem in China. Other formulations appear to be much safer. Perhaps one ongoing trial on its use to treat pulmonary hypertension [102] may provide us with a more definitive conclusion on this issue. Our data strongly suggest that TMP is a compound with promising therapeutic potential for treating endometriosis. Naturally, more investigation on its mechanism of action is warranted, and more clinical studies are badly needed.

Our study has several strengths. First, it used a recently established mouse model of deep endometriosis, which should recapitulate some most important features of the human condition, especially fibrosis [21]. Second, we tested a novel hypothesis that TMP can restrain lesional progression and fibrogenesis by inhibiting lesional aggregation of platelets, along with their subsequent profibrotic effects. The data are consistent with our hypothesis, and this should stimulate more research in this area in the future. Lastly, through testing the hypothesis by both in vitro and in vivo experimentations, our data provide not only correlational evidence but also direct evidence that TMP plays a role in attenuating lesional fibrogenesis by inhibiting the effect of platelets.

Of course, our study also has some limitations. First, while we have shown that TMP retards lesional progression and fibrogenesis through suppression of EMT and FMT, the exact underlying molecular mechanisms still remain uninvestigated. Second, we only evaluated the expression of TGF-β1, but not its upstream or downstream signaling pathway, to elucidate the molecular mechanisms of action for TMP. This, of course, is due mostly to the fact that we still do not know much about its upstream and downstream pathways. Third, we have evaluated the lesional staining of NK1R in our mouse experiment, but not in the in vitro experiment. Nor did we evaluate other markers of the NK1R signaling pathway, which might further illuminate the mode of action for TMP in treating endometriosis. Further research is warranted to further pin down the mechanisms of action for TMP.

In summary, our study has shown that TMP treatment stalls lesional progression and fibrogenesis through suppression of platelet activation, platelet-induced EMT, FMT, cellular contractility, and collagen production. Our in vivo experiment demonstrates that treatment with TMP significantly reduced lesion weight and the extent of lesional fibrosis, and alleviated hyperalgesia. Thus, our findings indicate that TMP could be an excellent drug candidate for treating endometriosis.

## Supplementary Information

Below is the link to the electronic supplementary material.

Supplementary file1 (DOCX 7341 KB)

## Data Availability

Data are available to those who send written request to the corresponding authors, detailing their purpose of intended use.
