# Supplementary material for: Tetramethylpyrazine Retards the Progression and Fibrogenesis of Endometriosis
Source: Reprod Sci. 2022 Jan 31;29(4):1170–87. doi: 10.1007/s43032-021-00813-x (PMC8907108; doi:10.1007/s43032-021-00813-x)
Supplement: Supplementary file 1 — Supplementary file1 (DOCX 7341 KB) [file 43032_2021_813_MOESM1_ESM.docx]

**Tetramethylpyrazine Retards the Progression and Fibrogenesis of Endometriosis**

**Shenghui Huang, Fengyi Xiao, Sun-Wei Guo, Tingting Zhang**

**Supplementary Materials**


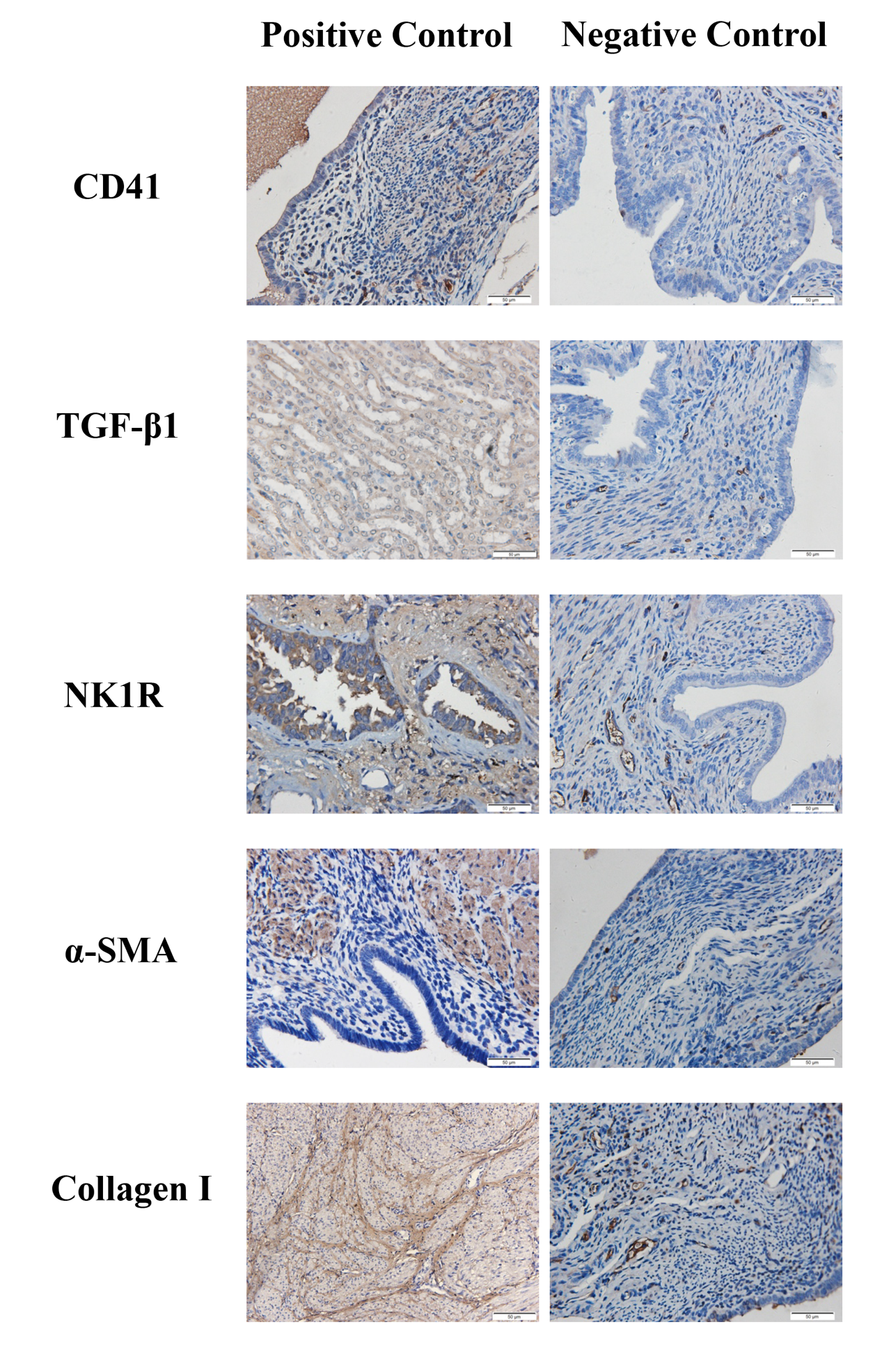


**Figure S1.** Positive and negative controls for immunostaining of CD41 (Ab33661), TGF-β1 (MAB240), NK1R (Ab183713), α-SMA (Ab5694) and collagen 1A1 (Ab292). For positive controls, mouse endometriosis tissues were used for CD41 staining^1^; mouse kidney tissues were used for TGF-β1 staining; human lung cancer tissues were used for NK1R; human endometriosis tissues were used for α-SMA staining; and human adenomyosis tissues were used for Collagen 1A1 staining. For negative controls, phosphate-buffered saline (PBS) was used. Scale bar = 50 µm.
